# Disorders of Sex Development of Adrenal Origin

**DOI:** 10.3389/fendo.2021.770782

**Published:** 2021-12-20

**Authors:** Gabriela P. Finkielstain, Ana Vieites, Ignacio Bergadá, Rodolfo A. Rey

**Affiliations:** ^1^ Centro de Investigaciones Endocrinológicas “Dr. César Bergadá” (CEDIE), CONICET – FEI – División de Endocrinología, Hospital de Niños Ricardo Gutiérrez, Buenos Aires, Argentina; ^2^ Universidad de Buenos Aires, Facultad de Medicina, Departamento de Biología Celular, Histología, Embriología y Genética, Buenos Aires, Argentina

**Keywords:** adrenal insufficiency, aldosterone, congenital adrenal hyperplasia, cortisol, DSD, glucocorticoid, lipoid, mineralocorticoid

## Abstract

Disorders of Sex Development (DSD) are anomalies occurring in the process of fetal sexual differentiation that result in a discordance between the chromosomal sex and the sex of the gonads and/or the internal and/or external genitalia. Congenital disorders affecting adrenal function may be associated with DSD in both 46,XX and 46,XY individuals, but the pathogenic mechanisms differ. While in 46,XX cases, the adrenal steroidogenic disorder is responsible for the genital anomalies, in 46,XY patients DSD results from the associated testicular dysfunction. Primary adrenal insufficiency, characterized by a reduction in cortisol secretion and overproduction of ACTH, is the rule. In addition, patients may exhibit aldosterone deficiency leading to salt-wasting crises that may be life-threatening. The trophic effect of ACTH provokes congenital adrenal hyperplasia (CAH). Adrenal steroidogenic defects leading to 46,XX DSD are 21-hydroxylase deficiency, by far the most prevalent, and 11β-hydroxylase deficiency. Lipoid Congenital Adrenal Hyperplasia due to StAR defects, and cytochrome P450scc and P450c17 deficiencies cause DSD in 46,XY newborns. Mutations in SF1 may also result in combined adrenal and testicular failure leading to DSD in 46,XY individuals. Finally, impaired activities of 3βHSD2 or POR may lead to DSD in both 46,XX and 46,XY individuals. The pathophysiology, clinical presentation and management of the above-mentioned disorders are critically reviewed, with a special focus on the latest biomarkers and therapeutic development.

## 1 Introduction

The term Disorders of Sex Development (DSD) refers to a wide range of anomalies occurring in the process of fetal sexual differentiation of the gonads and/or the genitalia, resulting in discordance between the chromosomal sex and the gonads and/or the internal and/or external genitalia.

### 1.1 The Physiology of Fetal Sex Differentiation

The chromosomal sex is determined at fertilization, depending on whether the spermatozoon carries an X or a Y chromosome. Nevertheless, during the first six weeks of embryogenesis in the human, there is no evidence of sex differences. This period is, therefore, called “undifferentiated” and is characterized by the existence of bipotential gonadal ridges, two sets of unipotential internal ducts –the Wolffian and the Müllerian ducts–, and bipotential urogenital sinus and primordia of external genitalia, in both the XX and the XY embryo.

**Figure 1:**
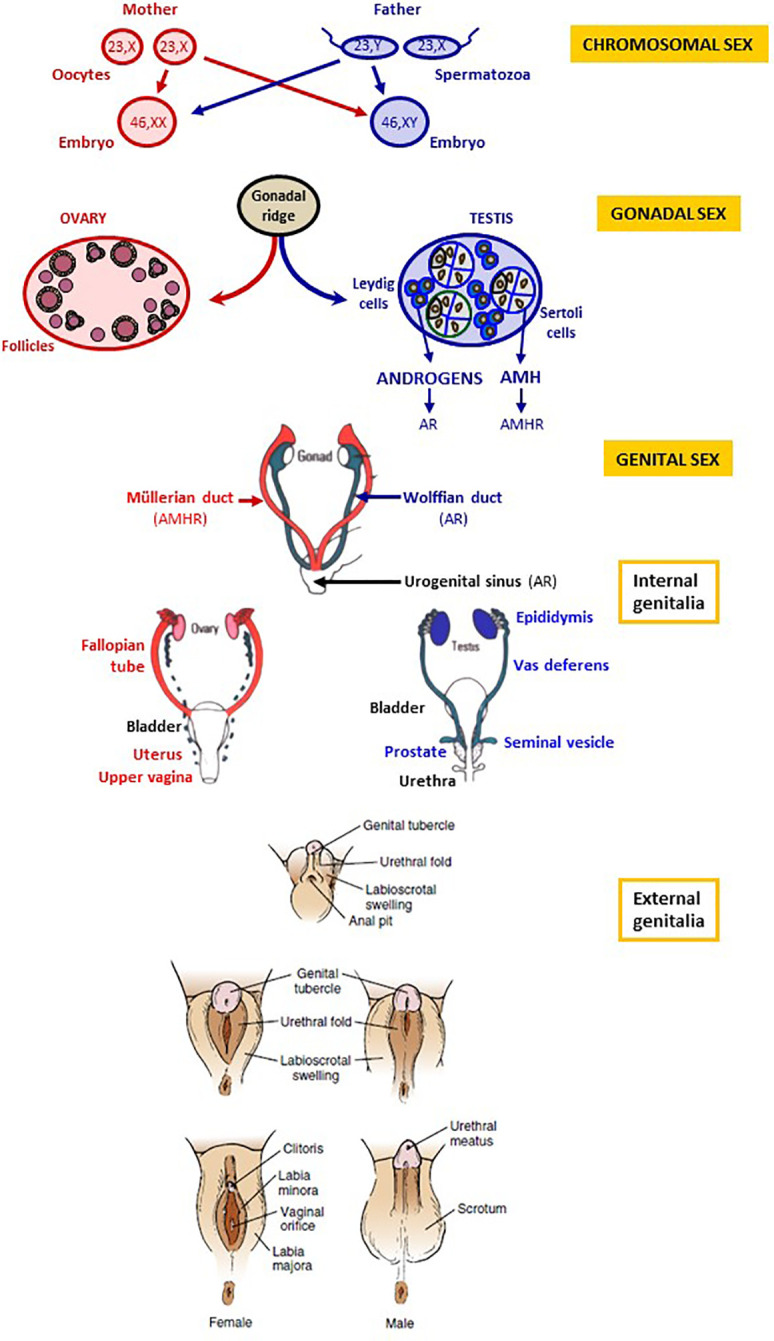
Chromosomal, gonadal and genital sex. Chromosomal sex is determined at fertilization, according to the X or Y chromosome carried by the spermatozoon. Gonadal sex differentiation occurs during the 7^th^ week of gestation: testes secrete androgens and anti-Müllerian hormone (AMH). The ovaries do not produce androgens and AMH in the first trimester of gestation. Genital differentiation is driven by testicular hormones: androgens produced by Leydig cells bind to the androgen receptor (AR) and induce the differentiation of the Wolffian ducts into the epididymides, the vasa deferentia and the seminal vesicles as well as the virilization of the urogenital sinus and of the external genitalia. In the absence of androgen action, the Wolffian ducts regress, and the urogenital sinus and the external genitalia undergo female differentiation. AMH, secreted by Sertoli cells, binds to the AMH receptor (AMHR) and provokes Müllerian duct regression; in the absence of AMH action, Müllerian ducts form the Fallopian tubes, the uterus and the upper vagina. Reproduced with permission from: Freire AV, Ropelato MG, Rey RA. Ovaries and Testes. In: Kovacs CS, Deal CL, editors. Maternal-Fetal and Neonatal Endocrinology: Physiology, Pathophysiology, and Clinical Management. Elsevier, 2020, pp 625-641. Copyright ^©^ 2000 Elsevier Inc (3).

During the 7^th^ week, the onset of the expression of SRY (Sex-determining region on the Y chromosome) in the XY embryo drives the indifferent gonad towards testicular differentiation by disrupting the existing balance between pro-testicular and pro-ovarian genes (1, 2). The testis secretes androgens and anti-Müllerian hormone (AMH), whose actions are critical in the process of genital differentiation (**Figure 1**). Androgens are responsible for Wolffian duct development into the epididymis, vas deferens and seminal vesicle, and the virilization of the urogenital sinus and the external genitalia. The urogenital sinus gives rise to the bladder, the proximal portion of the urethra and the prostate. The genital tubercle forms the penis, the labioscrotal folds differentiate into the scrotum and the urogenital folds fuse to form the penile urethra. The genital and the urinary systems flow into a single orifice. On the other hand, AMH induces the regression of the Müllerian ducts.

In the XX embryo, the ovaries do not produce androgens or AMH at this stage of development. Therefore, the Wolffian ducts regress, and the urogenital sinus and external genitalia follow the female pathway with no need for estrogen activity. The Müllerian ducts form the Fallopian tubes, the uterus and the upper part of the vagina. The urogenital sinus gives rise to the bladder, the urethra and the lower part of the vagina. The genital tubercle forms the clitoris, the labioscrotal folds differentiate into the labia majora and the urogenital folds into the labia minora. A detailed description of the physiology and the molecular and cellular biology of sex differentiation in mammals is available elsewhere (4).

### 1.2 Pathogenesis of DSD

It is simple to understand that physiologically abnormal gonads containing dysgenetic testicular and/or ovarian tissue may develop in fetuses with sex chromosome abnormalities, such as 46,XX/46,XY, 45,X/46,XY or other sex chromosome mosaicisms or chimerism. These are known as “sex-chromosome DSD”. However, DSD can also occur in individuals with typical 46,XX or 46,XY karyotypes. The underlying pathogenic mechanisms involve either an androgen excess in the XX fetus or a deficient testicular hormone activity in the XY fetus (5, 6).

#### 1.2.1 Virilization of the 46,XX Fetus

**Figure 2:**
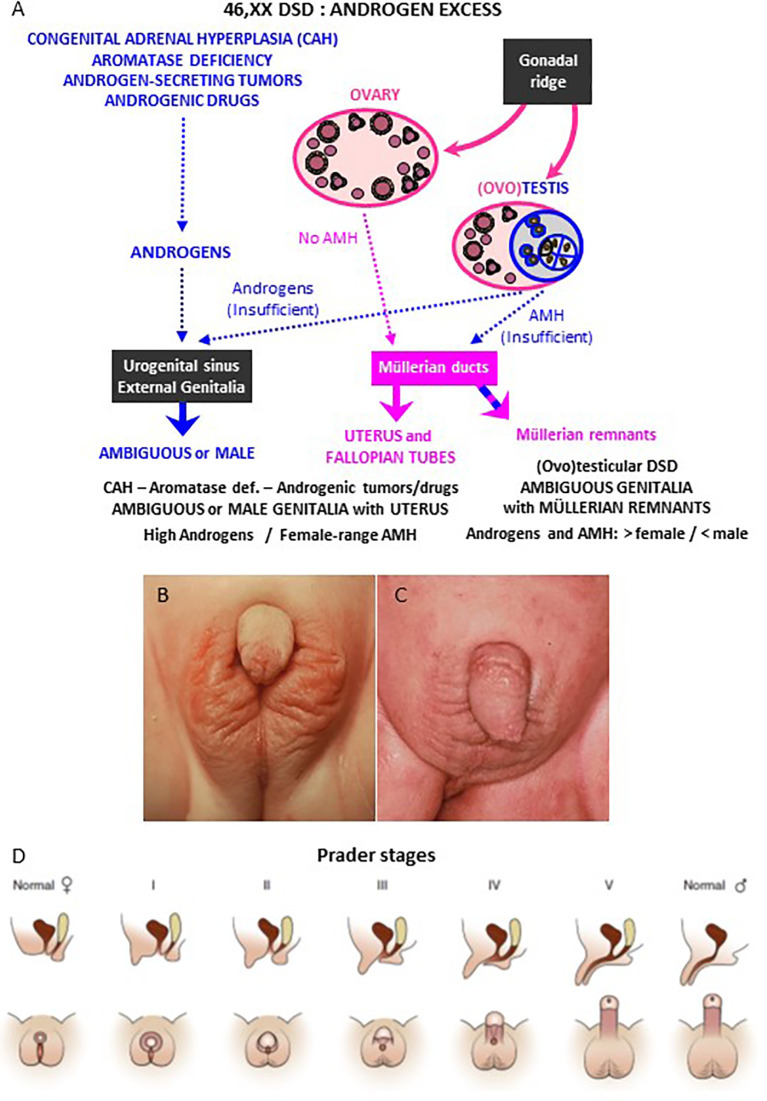
Genital virilization in 46,XX individuals. **(A)** Pathophysiology of virilization: virilization of external genitalia may occur in 46,XX patients with ovaries and hyperandrogenism of adrenal (congenital adrenal hyperplasia) or extra-adrenal (aromatase deficiency, androgenic tumors or drugs) origin; alternatively, virilization of external genitalia with partial regression of Müllerian ducts may occur in 46,XX patients with testicular or ovotesticular DSD. **(B**, **C)** External genitalia of 46,XX patients with congenital adrenal hyperplasia (CAH) due to 21-hydroxylase deficiency: Prader stage III **(B)** and stage V **(C)**. **(D)** Schematic of Prader staging for patients with CAH. Reprinted with permission from Rey RA, Josso N. Diagnosis and treatment of Disorders of Sexual Development. In: Jameson JL, De Groot LC, de Kretser DM, Giudice LC, Grossman A, Melmed S, Potts JT, Weir GC, eds. Endocrinology: Adult and Pediatric, 7th edition. Philadelphia: Elsevier Saunders; 2016:2086-2118. Copyright ^©^ 2016 Elsevier Inc (7). **(B**, **C)** kindly provided by Dr. M. Podestá, Buenos Aires, Argentina.

Excessive androgen action induces virilization of the XX fetus (**Figure 2A**). If there is exposure during the first trimester of intrauterine life, the final development of the external genitalia may be from completely male, when androgen levels are very high, to a milder virilization when androgen levels are lower. The different degrees of virilization have been classified by Prader in stages 1 to 5 (**Figures 2B–D**) (8). A later exposure to intrauterine androgens can no longer provoke a fusion of the labioscrotal folds but results in clitoris enlargement and labial swelling and rugosity.

**Figure 3:**
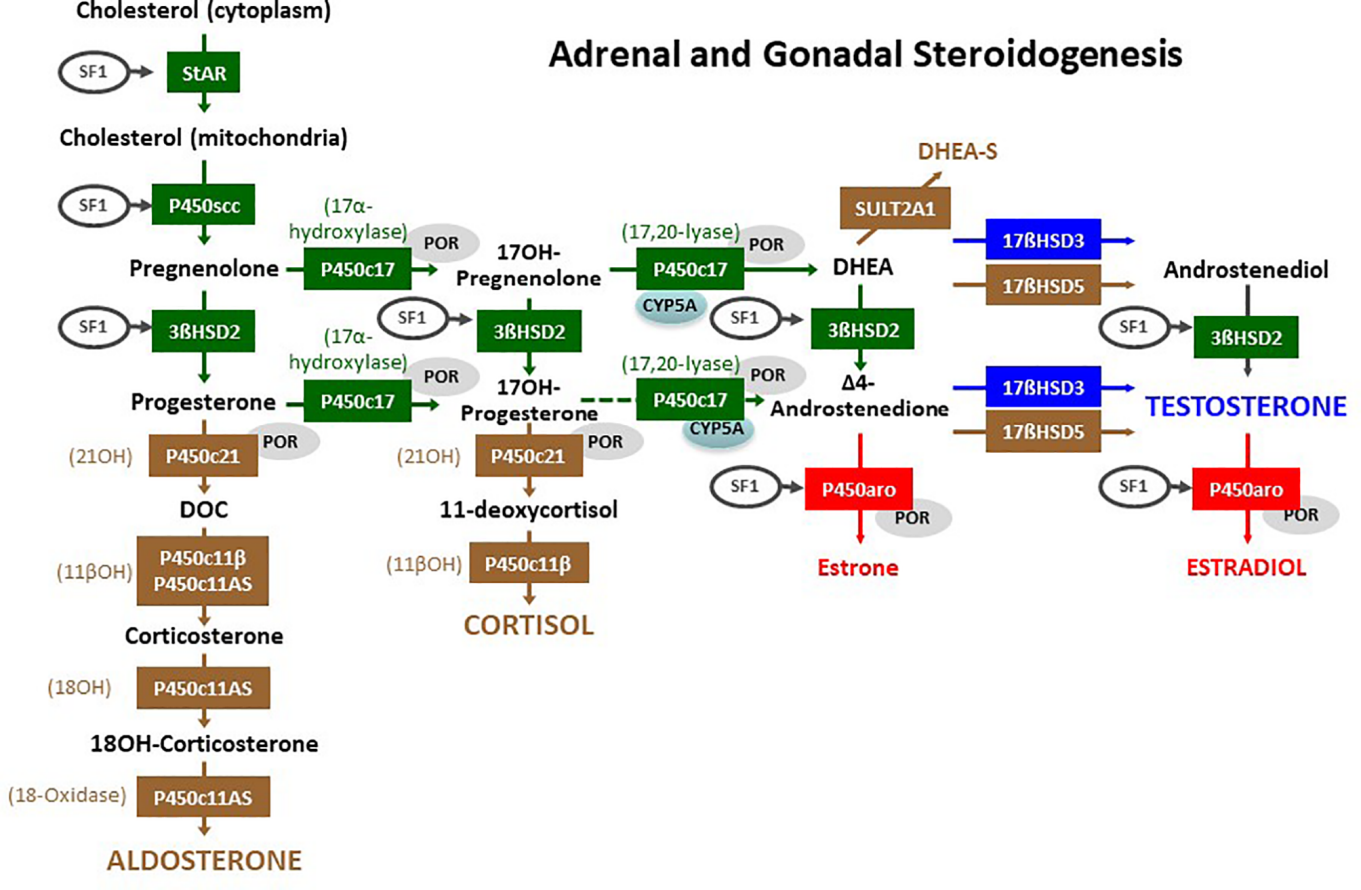
Adrenal and gonadal steroidogenesis. The initial steroidogenic steps (in green) are identical in the adrenals and the gonads. The steps in brown are specific of the adrenal cortex, and the steps in blue or red are specific of the gonads. Steroidogenic Acute Regulatory (StAR) protein enables cholesterol influx into the mitochondria. Cytochrome P450 side chain cleavage (P450scc) enzyme removes the cholesterol side chain yielding the first C21, Δ5-steroid pregnenolone. All Δ5-steroids are converted to Δ4-steroids by 3β-hydroxysteroid dehydrogenase type 2 (3βHSD2). In the zona glomerulosa of the adrenal cortex, the first Δ4-steroid progesterone, is converted to deoxycorticosterone (DOC) by the 21-hydroxylase (21OH) activity of cytochrome P450c21; subsequently, the 11β-hydroxylase (11βOH) activity of P450c11β (encoded by *CYP11B1*) or of the aldosterone synthase (P450c11AS, encoded by *CYP11B2*) catalyzes DOC conversion to corticosterone, and finally P450c11AS, through its 18-hydroxylase (18OH) and 18-methyl oxidase (18-oxidase) activities respectively yields 18-hydroxycorticosterone (18OH-corticosterone) and aldosterone. In the zona fasciculata, cytochrome P450c17 converts pregnenolone and progesterone to 17-hydroxypregnenolone (17OH-Pregnenolone) and 17-hydroxyprogesterone (17OH-Progesterone), which is subsequently converted to 11-deoxycortisol by 21OH and to cortisol by 11βOH. In the zona reticularis of the adrenal cortex and in the gonads, the 17,20-lyase activity of P450c17 is facilitated by cytochrome b5 (CYP5A) yielding dehydroepiandrosterone (DHEA) and only secondarily androstenedione. DHEA may be sulphated to DHEA-S by sulfotransferase 2A1 (SULT2A1) in the adrenal. Gonadal 17β-hydroxysteroid dehydrogenase (17βHSD) type 3 converts DHEA to androstenediol and androstenedione to testosterone; in the adrenal these steps are minorly catalyzed by 17βHSD type 5 (encoded by *AKR1C3*). In the ovary, cytochrome P450 aromatase (P450aro) converts androstenedione to estrone and testosterone to estradiol. The activity of many of these enzymes is induced by steroidogenic factor 1 (SF1, also known as AD4BP, encoded by *NR5A1*) or by the cytochrome P450 oxidoreductase (POR). Reproduced with modifications from: Rey RA, Grinspon RP. Normal male sexual differentiation and aetiology of disorders of sex development. Best Practice & Research Clinical Endocrinology & Metabolism (2011) 25:221-238. doi: 10.1016/j.beem.2010.08.013. Copyright ^©^ 2010 Elsevier Ltd (6).

Androgens may have different origins. Exaggerated production may arise from the adrenal cortex, that normally synthesizes androgens (**Figures 2A** and **3**), or from their lack of aromatization to estrogens by the placenta. Alternatively, androgen excess results from the existence of testicular tissue, in disorders such as ovotesticular or testicular DSD, or from maternal sources, such as adrenal or ovarian neoplasms, non-neoplastic disorders or androgenic drug use (**Figure 2A**).

##### 1.2.1.1 Androgens of Fetal or Fetoplacental Origin

Excessive androgen production from adrenal origin results from pathogenic variants in genes involved in steroidogenesis and encoding 21-hydroxylase, 11β-hydroxylase, 3β-hydroxysteroid dehydrogenase or P450 oxidoreductase. Congenital adrenal hyperplasia (by 21-hydroxylase alteration) is the commonest cause of virilization of the XX fetus, and it will be discussed in detail in this review.

Androgens of fetal gonadal origin may also be the cause of virilization *in utero* of 46,XX patients: the existence of dysgenetic testicular tissue, alone (46,XX testicular DSD) or associated with ovarian tissue (46,XX ovotesticular DSD).

Androgens are converted to estrogens through the action of the enzyme P450 aromatase (**Figure 3**). During pregnancy, the fetal component of the placenta expresses aromatase and is the major site of estrogen synthesis. Inactivating mutations in *CYP19A1*, encoding aromatase, result in an accumulation of fetal androgens that provoke the virilization of the XX fetus (9). Maternal virilization occurs during pregnancy but disappears progressively after delivery.

Adrenal function is not altered in patients with testicular/ovotesticular DSD or with placental aromatase deficiency; therefore, these conditions will not be further discussed in this review.

##### 1.2.1.2 Androgens of Maternal Origin

Virilization is notoriously milder when the excess of androgens is of maternal source because the placenta has a protective role by aromatizing them to estrogens (10). Nonetheless, some degree of virilization may occur in the 46,XX fetus if her mother suffered from androgen-secreting neoplasms, such as granulosa/theca cell tumors, thecomas and Sertoli-Leydig cell tumors of the ovary, or androgen secreting adrenal carcinomas and adenomas. In these cases, virilization of the mother persists until treatment, whereas virilization of the fetus partially regresses (10). Other non-neoplastic disorders characterized by androgen production are pregnancy luteomas and hyperreactio luteinalis (11). Since these are self-limited disorders of pregnancy, virilization wanes in both the mother and the newborn after birth.

#### 1.2.2 Undervirilization of the 46,XY Fetus

Insufficient testicular hormone action on target organs results in undervirilization of the XY newborn. When the lack of androgen action is complete, the newborn has an entirely female aspect of the external genitalia, and the condition may go undiagnosed until pubertal age (5, 6). The underlying etiologies can be classified into three groups: testicular dysgenesis, steroid synthesis defects and target organ defects.

Dysgenetic DSD is due to abnormalities in the process of testis differentiation (2), leading to a fetal-onset hypogonadism characterized by low or undetectable testosterone and AMH levels (12). The newborn has female or ambiguous external genitalia and persistence of Müllerian derivatives, i.e. uterus and Fallopian tubes.

A dissociated testicular dysfunction occurs in patients with normal AMH production but impaired androgen secretion (12). These patients have female or ambiguous external genitalia but do not have uterus and Fallopian tubes. The defect in androgen production may be limited to the testis, e.g. in Leydig cell hypoplasia due to mutations in *LHCGR*, the gene encoding the LH/hCG receptor, or in *HSD17B3*, which codes for 17β-hydroxysteroid dehydrogenase type 3, responsible for the conversion of androstenedione to testosterone (13). The other defects of androgen synthesis affect steroidogenic steps shared by the testis and the adrenal cortex (**Figure 3**) and will be described in detail in this review.

Finally, undervirilization of the XY fetus may result from defects in androgen target organs. Testicular androgen and AMH production is normal, but either dihydrotestosterone synthesis from testosterone is defective or the androgen receptor function is impaired (6).

## 2 DSD Associated With Adrenal Disorders

Congenital disorders affecting adrenal function may be associated with DSD in both 46,XX and 46,XY individuals, yet with a different underlying pathophysiology. While in 46,XX cases, the adrenal dysfunction is responsible for DSD, in 46,XY patients DSD results from the associated testicular dysfunction. In the vast majority of the cases, there is a primary adrenal insufficiency characterized by a reduction in cortisol secretion and overproduction of ACTH. In addition to cortisol deficiency, patients may exhibit different degrees of aldosterone deficiency leading to salt-wasting adrenal crises that can be severe and sometimes fatal. The trophic effect of ACTH provokes adrenal cortex hyperplasia, which justifies the denomination of congenital adrenal hyperplasia (CAH).

CAH is a group of autosomal recessive disorders resulting in defects in one of the proteins or enzymes involved in cortisol biosynthesis: steroidogenic acute regulatory protein (StAR), P450 cholesterol side-chain cleavage enzyme (P450scc), P450 17α-hydroxylase/17,20-lyase (P450c17), P450 oxidoreductase (POR), 3β-hydroxysteroid dehydrogenase type 2 (3βHSD2), P450 21-hydroxylase (21OH or P450c21), or 11β-hydroxylase (11βOH) (**Figure 3**). The first report of CAH dates from 1865, describing a man with female internal genitalia and enlarged adrenals who experienced sudden death (14). The various forms of CAH lead to different hormonal imbalances. Production of glucocorticoids, mineralocorticoids and sex steroids might be either compromised or, in some cases, normal. Most forms of CAH can be subdivided into classic (or severe), presenting at birth, and non-classic, diagnosed later in life because of mild hyperandrogenism leading to growth and bone age acceleration, precocious pubarche and increase in penile or clitoris size in childhood (14, 15).

DSD is present as a consequence of androgen excess in 46,XX or deficiency in 46,XY patients, according to the specific enzymatic defect and the severity of impairment (14). Adrenal steroidogenic defects leading to 46,XX DSD are 21-hydroxylase deficiency (21OHD), by far the most prevalent cause, and 11β-hydroxylase deficiency (11βOHD). On the other hand, Lipoid Congenital Adrenal Hyperplasia due to StAR defects, and P450scc and P450c17 deficiencies cause DSD in 46,XY newborns. Steroidogenic Factor 1 (SF1, also known as AD4BP) defects may also result in combined adrenal and testicular failure leading to DSD in 46,XY individuals. Finally, impaired 3βHSD2 and POR functions result in both 46,XX and 46,XY DSD.

### 2.1 46,XX DSD of Adrenal Origin

The common pathogenesis of DSD in 46,XX patients is the excessive androgen production by the adrenal cortex resulting from cortisol synthesis blockage (**Table 1**). The resulting increase in pituitary ACTH secretion, due to failure of the negative feedback, leads to the accumulation of cortisol steroid precursors that are derived to the androgen synthesis pathway (**Figure 3**).

**Table 1:** Distinctive features of DSD associated with adrenal dysfunction in 46,XX patients.

Protein Activity	Gene Chromosome	Transmission/Heterozygous carrier*	Mineralocorticoid pathway	Glucocorticoid pathway	Other steroids**	Signs of hyperandrogenism	Other features
P450c21 21-hydroxylase	*CYP21A2* 6p21.33	AR	DOC low Aldosterone low PRA high Sodium low Potassium high	Cortisol low 11-deoxycortisol low	Pregnenolone high 17OH-pregnenolone high Progesterone high 17OH-progesterone very high DHEA high Androstenedione very high Testosterone high 11-oxygenated androgens high	Prader I to V	Salt-wasting Hypoglycemia Hyperpigmentation Ovaries, uterus and Fallopian tubes present AMH in female range
P450c11β 11β-hydroxylase	*CYP11B1* 8q24.3	AR	DOC high Aldosterone low PRA low Sodium normal Potassium low/normal	Cortisol low 11-deoxycortisol high	Pregnenolone mildly high 17OH-pregnenolone mildly high Progesterone mildly high 17OH-progesterone mildly high DHEA mildly high Androstenedione mildly high Testosterone mildly high 11-oxygenated androgens mildly high	Prader I to V	Hypertension Hyperpigmentation Ovaries, uterus and Fallopian tubes present AMH in female range
3βHSD2 3β-hydroxysteroid dehydrogenase	*HSD3B2* 1p12	AR	DOC low Aldosterone low PRA high Sodium low Potassium high	Cortisol low 11-deoxycortisol low	Pregnenolone high 17OH-pregnenolone high Progesterone low 17OH-progesterone low DHEA high Androstenedione low Testosterone high	Prader I to III	Salt-wasting Hypoglycemia Hyperpigmentation Ovaries, uterus and Fallopian tubes present AMH in female range
POR P450 oxidoreductase, cofactor to P450scc, P450c17 and P450aro	*POR* 7q11.23	AR	DOC mildly high PRA mildly low Sodium normal/high Potassium normal/low	Cortisol mildly low 11-deoxycortisol mildly low	Pregnenolone mildly high 17OH-pregnenolone mildly high Progesterone high 17OH-progesterone high DHEA low Androstenedione low Testosterone mildly high	Prader I to IV	Hyperpigmentation Estradiol low Ovaries, uterus and Fallopian tubes present AMH in female range (rare) Hypertension Hypoglycemia

*Mode of transmission: AR, autosomal recessive. Heterozygous carrier: phenotype observed in heterozygous carriers of pathogenic gene variants.

**Steroid levels are considered low, normal or high as compared to reference values for females (46,XX chromosomal sex).

#### 2.1.1 21Hydroxylase Deficiency (21OHD)

##### 2.1.1.1 Pathophysiology and Clinical Presentation

The enzyme 21OH (P450c21) catalyzes the conversion of 17-hydroxyprogesterone into 11-deoxycortisol in the zona fasciculata and progesterone into 11-deoxycorticosterone (DOC) in the zona glomerulosa of the adrenal cortex. 21OHD (MIM 201910) due to mutations in *CYP21A2* (MIM 613815) represents the most common form, accounting for approximately 95% of CAH (16). *CYP21A2* and its highly homologous pseudogene *CYP21A1P* map to 6p21.3, about 30 kb apart. Due to the high homology in their sequences, mutations causing 21OHD typically occur from unequal recombination events between *CYP21A2* and *CYP21A1P*, abolishing enzymatic activity in different degrees (17). The estimated incidence, based on neonatal screening programs, ranges between 1/14,000 to 1/18,000 live births (14). Prevalence of heterozygous carries is around 1/60 (15).

**Figure 4:**
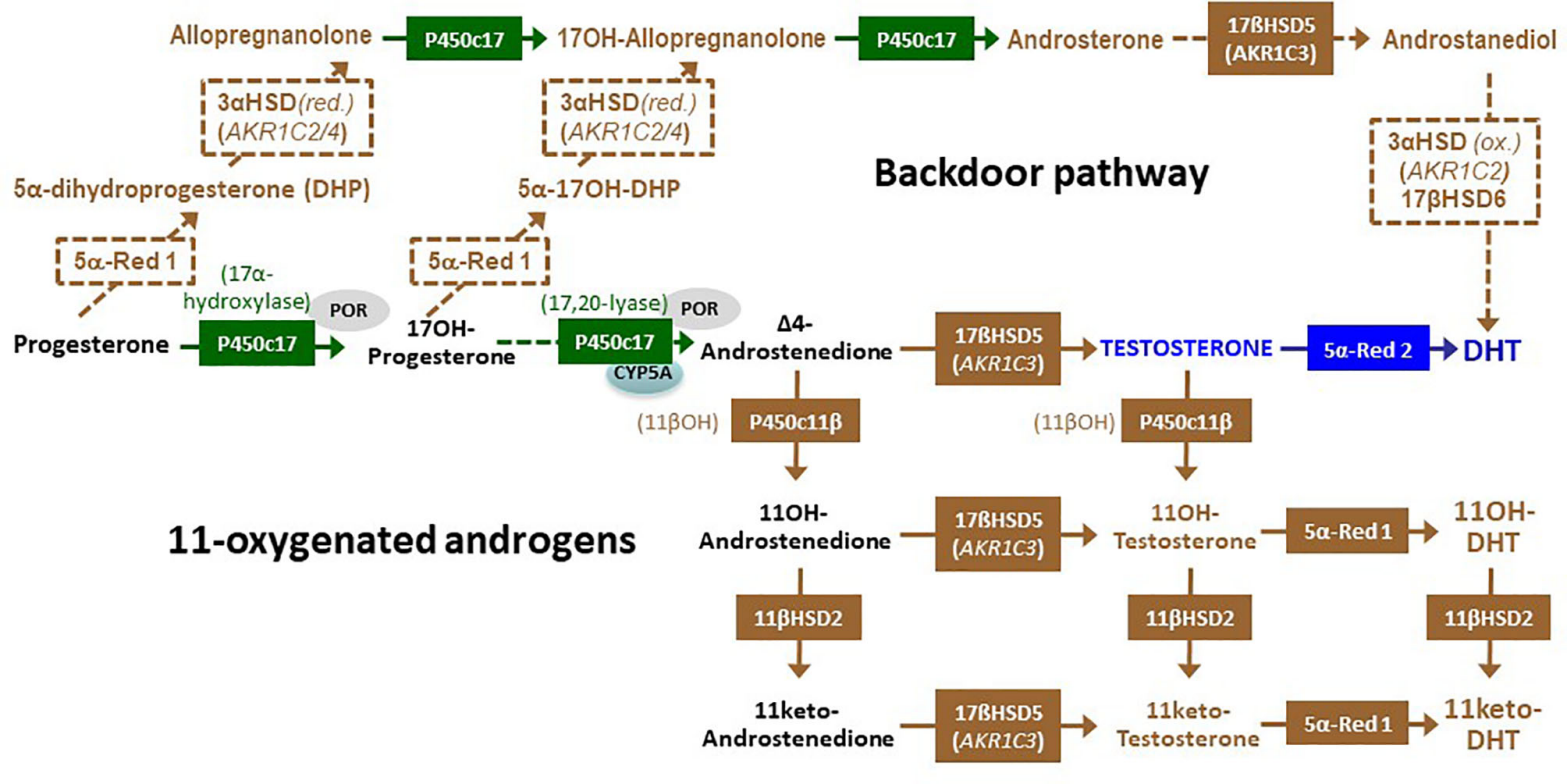
The alternative or “backdoor” pathway of androgen synthesis and the 11-oxygentaed androgens. In the “backdoor” pathway, progesterone is converted to 5α-dihydroprogesterone (DHP) by 5α-reductase type 1 (5α-Red 1) and subsequently reduced to allopregnanolone by 3α-hydroxysteroid dehydrogenase (3α-HSD) encoded by either *AKR1C2* or *AKR1C4*. Similarly, 17-hysroxyprogetserone (17OH-progesterone) is converted to 5α-17-hydroxy DHP and to 17hydroxy-allopregnanolone (17OH Allopregnanolone). Cytochrome P450c17, though its 17,20 lyase activity, catalyzes 17OH Allopregnanolone conversion to androsterone. Subsequently, androsterone can be metabolized by adrenal 17β-hydroxysteroid dehydrogenase type 5 (17βHSD5) to yield androstanediol, which is oxidized by 3α-HSD encoded by *AKR1C2* and finally transformed to dihydrotestosterone (DHT) by 17βHSD type 6 (also known as retinol dehydrogenase or RoDH), without involving testosterone. Blockage of cortisol synthesis resulting in the accumulation of 17-hydroxyprogesterone and androstenedione leads to the synthesis of 11-oxygenated androgens: the 11β-hydroxylase activity of cytochrome P450c11β catalyzes the conversion of androstenedione and testosterone to 11-hydroxyandrostenedione (11OH-androstenedione) and 11-hydroxytestosterone (11OH-testosterone), respectively. Subsequently, 11β-hydroxysteroid dehydrogenase type 2 (11βHSD2) converts them to 11keto-androstenedione and 11keto-testosterone. Finally, 5α-Red 1 leads to the synthesis of 11-hydroxy-DHT and 11-keto-DHT.

21OHD shows a wide spectrum of phenotypes, no longer representing a clear cut between the classic and non-classic forms, as historically reported, but depicting a continuum between both forms which depends on the remaining enzyme activity (15, 18). Classic CAH is the most severe form, and it is currently the most common cause of DSD and of primary adrenal insufficiency during childhood (15, 18). Inadequate cortisol production leading to increased ACTH secretion results in accumulation of steroid precursors upstream of 21OH action, namely progesterone and 17-hydroxyprogesterone, which are derived to the adrenal androgen pathway *via* the “classic” (**Figure 3**) and “backdoor” (**Figure 4**) pathways. Consequently, affected 46,XX fetuses experience virilization of external genitalia in early stages of development. There is a failure of separate vaginal formation, with the urogenital sinus emptying into the urethra leading to a single opening of the urinary and reproductive tracts, like in the male. Genital tubercle trophism is stimulated by androgens resulting in clitoral enlargement, whereas labioscrotal folds become more or less fused. Different degrees of virilization are quantified by a scale ranging from I to V, developed by Prader (**Figure 2D**). Hyperpigmentation is one of the clinical features that 21OHD cases may present due to hypersecretion of ACTH in the fetal stage. At variance with external virilization, normal uterine development derived from Müllerian structures is observed internally, owing to normally absent AMH production by the ovaries in the first trimester of fetal life (**Figure 2A**).

In the classic salt wasting form of 21OHD, residual enzymatic activity is less than 1%, with both cortisol and aldosterone deficiencies resulting in life-threatening adrenal crises in the first 2 weeks of life, which can be anticipated if neonatal screening for CAH is performed. Simple virilizing forms retain about 1-2% of enzymatic activity; therefore, there is minimal but sufficient aldosterone production to prevent salt wasting crises. However, because all patients have some degree of salt-wasting, and clinical presentation overlap, this subclassification is no longer fully reliable.

In childhood, pseudoprecocious and central precocious puberty, advanced bone age and impaired final height -are common features of the disease as a consequence of periods of hyperandrogenism and long-term glucocorticoid treatment (15). Later in life, common long-term complications in female adolescents and young adults with CAH include pubertal dysfunction, menstrual abnormalities, and fertility issues (19, 20). Pathophysiology of these complications includes an excess of C19 steroids of classic adrenal steroidogenesis and of the more recently explored alternative or “backdoor” pathway involving 11-oxo-steroids (21, 22). High levels of 17-hydroxyprogesterone and progesterone play a role in menstrual abnormalities: serum progesterone >0.6 ng/mL in the follicular phase leads to reduced LH pulse frequency and amplitude, and oligo/amenorrhea (23, 24). Fertility is impaired, especially in salt-wasting forms, but pregnancy and delivery rates are not reduced (23, 25). Potential causes of reduced fertility include anatomical issues due to hyperandrogenism, unfavorable cervical mucus for sperm migration, and endometrial thickening impairing embryo implantation and psychosocial factors (24). Ovarian adrenal rest tumors (OART) are rare in females with CAH, in contrast to the higher prevalence of testicular adrenal rest tumors (TART) present in males and are usually secondary to longstanding poor hormonal control (20).

##### 2.1.1.2 Diagnosis

Diagnosis of 21OHD in 46,XX newborns is based on a positive neonatal screening in those countries where this procedure is established, or by elevated serum levels of 17-hydroxyprogesterone, typically above 1000 ng/dl (**Table 1**). Screening laboratories are expected to employ a second-tier screen by mass spectrometry, which is preferred to other methods such as genetic studies; immunoassays have a higher rate of false-positive results. If liquid chromatography–tandem mass spectrometry is not available, occasionally an ACTH stimulation test is recommended to distinguish 21OHD from other adrenal steroidogenic defects, especially in individuals with borderline 17-hydroxyprogesterone (26). The use of 11-deoxycortisol may show advantages and avoid false positive results sometimes observed with 17-hydroxyprogesterone (27). Other laboratory findings, such as elevated testosterone and androstenedione with normal female levels of gonadotropins and AMH, are not needed to certify the diagnosis. In childhood, the diagnostic criteria are the same.

Life-threatening salt-wasting forms, representing about 75% of classic CAH, are generally due to gene deletions or conversions, stop codons, frame shifts or variants severely affecting 21OH activity, thus impairing both glucocorticoid and mineralocorticoid synthesis. The clinical signs of salt loss, i.e. low sodium and elevated potassium, are usually seen between days 5 and 15 after birth. Simple virilizing forms are usually associated with missense gene variants, which retain enough enzyme activity to produce the small amounts of aldosterone required to maintain salt balance. As mentioned, even those cases classified as simple virilizing may show a subclinical degree of aldosterone deficiency. Although genetic testing searching for *CYP21A2* variants is not used as the first-line diagnostic test, genotyping is key for establishing affected carriers in the family (15).

#### 2.1.2 11β-Hydroxylase Deficiency (11βOHD)

##### 2.1.2.1 Pathophysiology and Clinical Presentation

The microsomal cytochrome P450c11β, with 11β-hydroxylase activity, is encoded *CYP11B1* (MIM 610613) and catalyzes one of the final steps in cortisol biosynthesis: the conversion of 11-deoxycortisol (S compound) and 11-deoxycorticosterone (DOC) to cortisol and corticosterone, respectively (**Figure 3**). Mutations in *CYP11B1* gene cause 11βOHD (MIM 202010), the second most common form of CAH accounting for 0.2-8% of all cases. The estimated prevalence of this condition is 1 in 100,000 births, with higher prevalence in Muslim and Moroccan Jewish Middle Eastern populations (28). Impairment in both cortisol and corticosterone production causes increased ACTH secretion with accumulation of 11-deoxycortisol and DOC, respectively, which are shunted to the androgen pathway causing different degrees of virilization in affected females (29). Compared to females with 21OHD, those with 11βOHD are more virilized; intriguingly, the extent of masculinization, however, correlates poorly with the degree of hyperandrogenemia (30).

In childhood, persistent androgen excess may result in pseudoprecocious puberty, rapid somatic growth and accelerated bone maturation leading to premature epiphyseal closure and short stature (31). Later in life, hyperandrogenism results in delayed menarche. Lower fertility rates have been reported. So far, there is one report of pregnancy in a 26-year-old woman with severe 11βOHD deficiency (32).

Mild to moderate hypertension is present in two-thirds of patients with classic 11βOHD. Despite 11-deoxycorticosterone being a less potent mineralocorticoid than aldosterone, its accumulation causes salt retention and hyporeninemic hypokalemic hypertension, mainly in older children and adults. However, as newborns are relatively resistant to mineralocorticoids, salt loss might be present, but it is usually mild and transient (33).

Rare cases of non-classic 11βOHD have been described, presenting later in life with milder virilization, precocious pseudopuberty, hirsutism or menstrual irregularities.

##### 2.1.2.2 Diagnosis

Diagnosis of 11βOHD is based on elevated basal concentrations of DOC and hyperresponsiveness of 11-deoxycortisol during ACTH test (>3 times the upper limit of normal) (**Table 1**). Low cortisol and normal or suppressed plasma renin activity is also present (14). Nevertheless, 11βOHD diagnosis may be challenging in neonates, due to several reasons. Newborns often do not present with hypertension and suppressed renin. Another potential source of error is the mild to moderate elevations of 17-hydroxyprogesterone often observed, leading to an erroneous diagnosis of 21OHD deficiency. Lastly, in case deoxycorticosterone and 11-deoxycortisol are not specifically measured, the diagnosis may be missed. Molecular genetic testing confirms the diagnosis of 11βOHD when mutations in *CYP11B1* gene are found.

### 2.2 46,XY DSD Associated With Adrenal Dysfunction

As already mentioned, DSD in 46,XY patients do not result from adrenal failure, but from hypoandrogenemia due to the associated testicular steroidogenic defect (**Figure 3** and **Table 2**).

**Table 2:** Distinctive features of DSD associated with adrenal dysfunction in 46,XY patients.

Protein Activity	Gene Chromosome	Transmission/Heterozygous carrier*	Mineralocorticoid pathway	Glucocorticoid pathway	Other steroids**	Genitalia and gonads***	Other features
StAR Mitochondrial cholesterol transfer	*STAR* 8p11.23	AR	DOC low Aldosterone low PRA high Sodium low Potassium high	Cortisol low 11-deoxycortisol low	Pregnenolone low 17OH-pregnenolone low Progesterone low 17OH-progesterone low DHEA low Androstenedione low Testosterone low	EG: from female to male with hypospadias No uterus Testes with lipoid degeneration of Leydig cells	Salt-wasting Hypoglycemia Hyperpigmentation Lipoid adrenal hyperplasia Clinical androgen deficiency at puberty AMH in male range
P450scc Cholesterol side-chain cleavage	*CYP11A1* 15q24.1	AR	DOC low Aldosterone low PRA high Sodium low Potassium high	Cortisol low 11-deoxycortisol low	Pregnenolone low 17OH-pregnenolone low Progesterone low 17OH-progesterone low DHEA low Androstenedione low Testosterone low	EG: from female to male with hypospadias No uterus Testes with impaired Leydig cell androgen synthesis	Salt-wasting Hypoglycemia Hyperpigmentation Clinical androgen deficiency at puberty AMH in male range
P450c17 17α-hydroxylase, 17,20-lyase	*CYP17A1* 10q24.32	AR	DOC high Aldosterone low PRA low Sodium normal/high Potassium low	Cortisol low 11-deoxycortisol low	Pregnenolone high 17OH-pregnenolone high Progesterone mildly high 17OH-progesterone mildly high DHEA low Androstenedione low Testosterone low	EG: from female to male with hypospadias No uterus Testes with impaired Leydig cell androgen synthesis	Hypertension Hypoglycemia Hyperpigmentation Clinical androgen deficiency at puberty AMH in male range
P450c17 Isolated 17,20-lyase	*CYP17A1* 10q24.32	AR	Not affected	Not affected	Pregnenolone mildly high 17OH-pregnenolone high Progesterone mildly high 17OH-progesterone high DHEA low Androstenedione low Testosterone low	EG: from clitoromegaly with some labial fusion to male with hypospadias No uterus Testes with impaired Leydig cell androgen synthesis	Clinical androgen deficiency at puberty AMH in male range
Cytochrome b5, type A Cofactor to 17,20-lyase	*CYB5A* 18q22.3	AR	Not affected	Not affected	Pregnenolone mildly high 17OH-pregnenolone high Progesterone mildly high 17OH-progesterone high DHEA low Androstenedione low Testosterone low	EG: from clitoromegaly with some labial fusion to male with hypospadias No uterus Testes with impaired Leydig cell androgen synthesis	Clinical androgen deficiency at puberty AMH in male range
POR P450 oxidoreductase, cofactor to P450scc, P450c17 and P450aro	*POR* 7q11.23	AR	DOC mildly high PRA mildly low Sodium normal/high Potassium normal/low	Cortisol mildly low 11-deoxycortisol mildly low	Pregnenolone mildly high 17OH-pregnenolone mildly high Progesterone high 17OH-progesterone high DHEA low Androstenedione low Testosterone low	EG: from female to male with hypospadias No uterus Testes with impaired Leydig cell androgen synthesis	Hyperpigmentation Clinical androgen deficiency at puberty AMH in male range (rare) Hypertension Hypoglycemia
3βHSD2 3β-hydroxysteroid dehydrogenase	*HSD3B2* 1p12	AR	DOC low Aldosterone low PRA high Sodium low Potassium high	Cortisol low 11-deoxycortisol low	Pregnenolone high 17OH-pregnenolone high Progesterone low 17OH-progesterone low DHEA high Androstenedione low Testosterone low	EG: from female to male with hypospadias No uterus Testes with impaired Leydig cell androgen synthesis	Salt-wasting Hypoglycemia Hyperpigmentation Clinical androgen deficiency at puberty AMH in male range
SF1 (AD4BP) Regulator of StAR, P450scc, 3βHSD2	*NR5A1* 9q33.3	AD/AR	(rare) DOC low Aldosterone low PRA high Sodium low Potassium high	(rare) Cortisol low 11-deoxycortisol low	Pregnenolone low 17OH-pregnenolone low Progesterone low 17OH-progesterone low DHEA low Androstenedione low Testosterone low	EG: from female to male with hypospadias Uterus or Müllerian remnants may be present Testes: variable degree of dysgenesis	Clinical androgen deficiency at puberty AMH low (rare) Salt-wasting Hypoglycemia Hyperpigmentation

*Mode of transmission: AD, autosomal dominant; AR, autosomal recessive. Heterozygous carrier: phenotype observed in heterozygous carriers of pathogenic gene variants.

**Steroid levels are considered low, normal or high as compared to reference values for males (46,XY chromosomal sex).

***EG, external genitalia.

#### 2.2.1 StAR and P450scc Deficiencies

##### 2.2.1.1 Pathophysiology and Clinical Presentation

StAR protein has a crucial role in facilitating the influx of cholesterol between the outer and the inner mitochondrial membranes; subsequently, P450scc enzyme, encoded by *CYP11A1* gene, catalyzes the conversion of cholesterol to pregnenolone, the first and rate-limiting step in the synthesis of all steroid hormones (34).

The pathophysiology of StAR and P450scc deficiencies is similar except that lipid droplet accumulation typical of Lipoid Congenital Adrenal Hyperplasia (LCAH) caused by StAR deficiency does not occur in P450scc deficiency (34). There is a severe impairment of steroidogenesis in adrenals and gonads, leading to minimal concentrations of all steroids. Adrenal insufficiency leads to failure to thrive, salt wasting due to aldosterone deficiency, hypoglycemia due to cortisol deficiency, and consequent elevation of ACTH and plasma renin activity (33). Testicular failure is limited to Leydig cell dysfunction, with hypoandrogenism leading to defective virilization of the Wolffian ducts, the urogenital sinus and the external genitalia. Conversely, because AMH is normally produced by Sertoli cells in early fetal life, there is no uterus or Fallopian tubes (6)..

A distinctive feature in the pathophysiology of StAR deficiency is explained by the “two-hit disease model” (35): the first hit is the absence of StAR, which reduces cholesterol import and, therefore, adrenal and testicular steroidogenesis. However, a small amount of steroidogenesis remains by StAR-independent mechanisms. The second hit occurs when the newly synthesized intracellular cholesterol, cholesterol esters and their autoxidation products progressively accumulate in lipid droplets, leading to grossly enlarged adrenals, and destroy residual StAR-independent steroidogenic mechanisms (33). Leydig cell destruction early in gestation causes deficient testosterone production. As expected, fetal sex development of 46,XX individuals is not altered; these patients are born with normal female genitalia, and most of them enter puberty normally due to StAR independent steroidogenesis. However, later in adolescence gonadotropic stimulus results in cellular damage affecting mainly the luteal phase, leading to irregular menses (33). Low levels of estradiol might be insufficient for embryo implantation, resulting in infertility (25). Thus far, pregnancy has been reported in three women with a StAR gene mutation who presented with spontaneous puberty and menarche. These pregnancies were achieved using reproductive technology: clomiphene citrate in one and IVF in the remaining two patients (36–38).

LCAH (MIM 201710) is the most severe form of CAH, caused by pathogenic variants in the *STAR* gene (MIM 600617) (35). Despite being a rare defect, LCAH is more frequently seen in certain populations, such as East Asian, Arab and Swiss due to the presence of mutations with founder effect. For example, mutation Q258X was found in more than 70% of affected alleles in Japan and Korea representing about half of all reported cases (39). So far, more than 40 mutations have been described in 190 patients (34, 40, 41). P450scc deficiency (MIM 613743), due to *CYP11A1* mutations (MIM 118485), is a rare disorder that can present at any time, from infancy to early childhood. To date, less than 40 cases have been reported (14).

Typically, 46,XY affected infants are born with female or ambiguous genitalia and present with neonatal adrenal crises. Hyperpigmentation is frequent, associated with elevation of ACTH. Affected patients are generally raised as girls (34).However, a number of cases with mild forms resulting in normal male genitalia and late-onset adrenal insufficiency have also been reported (42). In these cases, the presence of testicular adrenal rest tumors (TART) has been described, leading to primary testicular failure with oligospermia and elevated FSH (43).

##### 2.2.1.2 Diagnosis

The diagnosis of DSD due to StAR or P450scc deficiency is suspected in a 46,XY newborn, phenotypically female or with ambiguous, hyperpigmented genitalia and failure to thrive in the first weeks of life. All gonadal and adrenal steroids are very low, ACTH, renin and LH are elevated, and AMH levels are within the normal range for chromosomal sex (**Table 2**). The differential diagnosis with other steroidogenic defects is based on the low levels of all steroids. In 46,XY patients with female or ambiguous genitalia, hyperpigmentation, absence of uterus in ultrasonography, male-range AMH levels and low levels of adrenal steroids with ACTH elevation distinguishes StAR and P450scc deficiencies from Leydig cell hypoplasia (6, 34). The differential diagnosis between StAR and P450scc deficiencies is limited to sequencing of *STAR* and *CYP11A1* genes. The enlarged adrenal size usually observed in LCAH is not seen in P450scc deficiency. However, adrenal size alone cannot distinguish both conditions (34).

#### 2.2.2 17α-Hydroxylase, 17,20-Lyase Deficiency (P450c17D)

##### 2.2.2.1 Pathophysiology and Clinical Presentation

P450c17 is a microsomal P450 enzyme expressed in both adrenals and gonads that catalyzes two major reactions in the steroidogenic pathway: the 17α-hydroxylation followed by the 17,20-lyase reactions resulting in the synthesis of 17α-hydroxylated glucocorticoids and sex steroids by the adrenal glands and gonads, respectively (33). Complete P450c17D (MIM 202110) is a rare form of CAH accounting for 1% of the cases, caused by mutations in *CYP17A1* gene (MIM 609300). To date, just over 100 mutations have been reported, some of them being more frequent in certain populations such as Dutch Friedlaenders, Southeast Asian and Brazilians, due to mutations with founder effect (44).

Steroidogenesis in adrenals and gonads is severely impaired, causing deficiency of cortisol and sex steroids, with mineralocorticoid excess. Consequently, 46,XY fetuses are severely undervirilized while 46,XX sexual development is unaffected at birth (33). The typical presentation of this form of CAH is a phenotypic girl or adolescent with pubertal failure, including lack of breast development and primary amenorrhea, hypertension and hypokalemia (45). Alternatively, like in all other forms of DSD of adrenal origin, 46,XY individuals may present with ambiguous genitalia and testes present in the inguinal canals or intra-abdominally. In contrast with most forms of CAH, patients with P450c17D do not develop adrenal crises despite low cortisol levels, because corticosterone has glucocorticoid activity and mineralocorticoid synthesis is unaffected (33). Manifestations of mineralocorticoid excess due to the accumulation of DOC, such as hypertension and hypokalemia, usually appear later in childhood due to the relative kidney insensitivity to mineralocorticoids present in infancy.

##### 2.2.2.2 Diagnosis

Like for other forms of steroid synthesis defects, DSD due to P450c17D are suspected in 46,XY girls or patients with ambiguous genitalia, absent uterus, testosterone above the female range but below the male range and AMH in the male range (**Table 2**). The distinctive feature of P450c17D is the elevation of pregnenolone, progesterone, DOC and corticosterone, associated to normal/low aldosterone and normal/low plasma renin activity, and decreased levels of steroids downstream P450c17 activity, i.e. 17-hydroxypregnenolone, 17-hydroxyprogesterone, 11-deoxycortisol and cortisol, as well as DHEA and androstenedione (33, 45). ACTH stimulation test may be necessary to evidence an increase in pregnenolone/17-hydroxypregenolone and progesterone/17-hydroxyprogesterone ratios (44). At pubertal age, gonadotropins are usually elevated reflecting gonadal dysfunction (45). Genetic analysis of *CYP17A1* confirms the diagnosis.

#### 2.2.3 Isolated 17,20-Lyase Deficiency

##### 2.2.3.1 Pathophysiology and Clinical Presentation

Isolated 17,20-lyase deficiency (MIM 202110) is a rare cause of CAH caused by mutations in any of three different genes: *CYP17A1*, *POR* or *CYB5A* (33). Missense mutations in *CYP17A1* (MIM 609300) affecting the redox partner binding site of the enzyme do not impair 17α-hydroxylase activity (44). The 17,20-lyase activity of P450c17 is also critical in the ‘backdoor’ pathway of dihydrotestosterone synthesis, through androstanediol, without going through DHEA, androstenedione and testosterone (**Figure 4**) (46, 47). Like other P450 enzymes, e.g. P450c21 (21OH) and P450aro (aromatase), P450c17 receives electrons from NADPH *via* P450 oxidoreductase (POR). Particularly human 17,20-lyase activity is stimulated by cytochrome b5 type A, acting as an allosteric factor. Therefore, impaired 17,20-lyase activity also results from mutations in *POR* (MIM 124015) and *CYB5A* (MIM 613218) (33, 44). POR defects result in a combined deficiency of 17,20-lyase, 21OH and aromatase, therefore likely to induce DSD in both 46,XX and 46,XY individuals; they will be addressed in a specific section below. Cytochrome b5 also reduces methemoglobin (ferric hemoglobin) to normal hemoglobin (ferrous hemoglobin); its defects result in associated methemoglobinemia (MIM 250790).

Clinically, 46,XY patients with 17,20-lyase deficiency present at birth with ambiguous genitalia, whereas 46,XX patients are usually detected when seeking attention for pubertal failure and primary amenorrhea. Infertility is the rule, and the first case of successful pregnancy and delivery in a 24-year-old woman after controlled ovarian stimulation and *in vitro* fertilization, has only recently been reported (48).

##### 2.2.3.2 Diagnosis

Isolated 17,20-lyase deficiency leads to insufficient virilization of 46,XY fetuses and normal genitalia in 46,XX fetuses. Failure to enter puberty, primary amenorrhea and infertility the most common clinical presentation in females.

Biochemically, the blockage of 17,20-lyase activity due to *CYP17A1*, *POR* or *CYB5A* mutations leads to marked elevation of 17OH-pregnenolone and pregnenolone and mild elevation of progesterone and 17OH-progesterone, with low levels of DHEA, androstenedione and testosterone, low C19 steroids and poor response to hCG (33).

#### 2.2.4 SF1 Defects

##### 2.2.4.1 Pathophysiology and Clinical Presentation

SF1, encoded by *NR5A1* (MIM 184757), was first described as a key regulator of the P450 steroid hydroxylases in the adrenals and gonads (**Figure 3**), and subsequently found to be involved in embryonic morphogenesis of the ventromedial hypothalamic nucleus, the gonadotropes, the adrenal cortex and the testes and ovaries (49, 50). Pathogenic variants found in *NR5A1* are associated with DSD in 46,XY individuals due to testicular failure during early fetal development (MIM 612965); in some cases, primary adrenal insufficiency is associated (51).

Clinically, 46,XY patients with SF1 defects present with variable degrees of undervirilization of the external genitalia, reflecting androgen deficiency, and of persistence of Müllerian derivatives, indicating AMH deficiency associated with testicular dysgenesis (**Table 2**). Adrenal failure occurs in a minority of the cases, with glucocorticoid and mineralocorticoid deficiencies.

In 46,XX individuals, ovarian dysgenesis leading to primary ovarian insufficiency has been described (MIM 612964), but as expected does not result in DSD. Recently, variants in *NR5A1* have been reported in virilized 46,XX patients with testicular or ovotesticular DSD (MIM 617480) (52); however, adrenal function does not seem affected.

##### 2.2.4.2 Diagnosis

This is the only form of 46,XY DSD where AMH deficiency exists together with adrenal dysfunction. Therefore, apart from low androgen and high LH levels, low AMH and high FSH levels should alert of a SF1 defect in a patient with ambiguous genitalia and Müllerian remnants associated with adrenal insufficiency. The detection of a mutation in *NR5A1* confirms the diagnosis (51).

### 2.3 Adrenal Disorders Causing 46,XX and 46,XY DSD

#### 2.3.1 3β-Hydroxysteroid Dehydrogenase Type 2 Deficiency (3βHSD2D)

##### 2.3.1.1 Pathophysiology and Clinical Presentation

Classic 3β-hydroxysteroid dehydrogenase type 2 deficiency (3βHSD2D) is a rare form of CAH with estimated incidence < 1/1,000,000 live births, accounting for less than 0.5% of all cases of this condition (53). Two functional *HSD3B* genes are found in humans: *HSD3B1* encodes an isozyme expressed in peripheral tissue including brain, liver, skin, mammary glands and placenta, and *HSD3B2* encodes 3β-hydroxysteroid dehydrogenase type 2 found in the adrenals and gonads. This isoenzyme normally converts Δ5-steroids (pregnenolone, 17-hydroxypregnenolone, dehydroepiandrosterone and androstenediol) to the corresponding Δ4-steroids (progesterone, 17-hydroxyprogesterone, androstenedione and testosterone). Classic 3βHSD2D (MIM 201810) is caused by *HSD3B2* gene mutations (MIM 613890) and characterized by impaired steroidogenesis in both adrenals and gonads. Consequently, cortisol, aldosterone, and androstenedione concentrations are low and renin, ACTH, and dehydroepiandrosterone (DHEA) concentrations are increased with DHEA being converted to testosterone by extra-adrenal 3βHSD1. Clinical features include ambiguous genitalia in both 46,XX and 46,XY fetuses and adrenal insufficiency of both glucocorticoids and mineralocorticoids (54).

Genotypic females are generally born mildly virilized, presenting with enlarged clitoris, incomplete labial fusion and genital hyperpigmentation due to the shift from DHEA to testosterone by HSD3B1 (**Table 1**); however, they can present with normal external genitalia at birth. Preserved mineralocorticoid function and non-virilized genitalia may lead to underdiagnosis (55). Genotypic males are invariably undervirilized due to insufficient testicular conversion of DHEA to testosterone (**Table 2**). Phenotypic manifestations include severe hypospadias, micropenis, bifid scrotum, and undescended testis (53). There is no correlation between the impairment in male sexual differentiation and salt- wasting (54). Some patients experience spontaneous puberty while others fail to progress through puberty needing sex hormone replacement (53).

In adult 46,XX patients, hyperandrogenism becomes challenging due to both the increasing androgen production by the zona reticularis and the increased conversion of testosterone to DHT (56). In males, TART and gonadal dysfunction, leading to arrested spermatogenesis and azoospermia, have been reported which warrants the need of long-term follow-up of these patients through their lifespan. Very limited information exists regarding fertility in both females and males with 3βHSD2D (19, 25, 53).

Non-classic 3βHSD2D was originally suspected in children with premature pubarche and in young females with hirsutism and menstrual irregularities who presented exaggerated Δ5-steroid production after ACTH stimulation and elevated 17-hydroxyprogesterone to cortisol ratio. Alternatively, this group of patients is referred to as having “partial 3βHSD2D” (33). Interestingly, genetic testing was unable to identify mutations in *HSD3B2* gene in all these patients, which raises doubts about the real existence of non-classic forms (14).

##### 2.3.1.2 Diagnosis

Primary biochemical abnormality in 3βHSD2D is the elevated Δ5 to Δ4 steroid ratio, including 17-hydroxypregnenolone/17-hydroxyprogesterone and DHEA/androstenedione ratios in serum, and pregnanetriol to pregnanediol ratio in urine, especially after ACTH stimulation. Diagnosis of the classic form of 3βHSD2D based on 17-hydroxypregnenolone levels above 100 nmol/L (3300 ng/dl) either basal or after ACTH stimulation is the best single biological criterion of 3βHSD2D. In addition, the baseline 1000-fold elevation of 17-hydroxypregnenolone to cortisol ratio and low 11-oxygenated androgens by liquid chromatography-tandem mass spectrometry (LC-MS/MS) provides an unequivocal biochemical diagnostic parameter (53, 55). Nonetheless, diagnosis at birth could be challenging due to HSD3B1 activity which can convert some of the elevated 17-hydroxypregnenolone to 17-hydroxyprogesterone, leading to false positives on neonatal screening for 21OHD (57). Genetic testing for *HSD3B2* mutations confirms the diagnosis of the classic form (53, 55).

#### 2.3.2 POR Deficiency (PORD)

##### 2.3.2.1 Pathophysiology and Clinical Presentation

As mentioned, POR is an important electron donor from NADPH to microsomal P450 enzymes, such as 17α-hydroxylase, 21-hydroxylase and P450 aromatase (33). POR deficiency (MIM 613571) due to mutations in the *POR* gene (MIM 124015) results in an unusual form of CAH first described in 2004, characterized by partially deficient P450c17 activity, with or without associated deficient activity of P450c21 and P450aro (58–60). Approximately 75 mutations have been reported to date in 140 individuals (16).

Due to the variability in enzymatic impairment, there is a wide spectrum of clinical phenotypes ranging from ambiguous genitalia in both 46,XX and 46,XY individuals with adrenal insufficiency to milder phenotypes in women who appear to have a form of polycystic ovary syndrome, or mildly affected men with gonadal insufficiency (33). Generally, 46,XY patients are born with undervirilization due to impaired of 17,20 lyase activity resulting in decreased androgen production (**Table 2**). On the other hand, 46,XX females present with virilized genitalia (**Table 1**), which depends on the causative *POR* mutation. One possible explanation is that certain mutations (e.g. R457H) affect placental P450aro activity leading to maternal and 46,XX fetal virilization during pregnancy due to defective conversion of fetal adrenal C19 androgen precursors to estrogens (33, 61). An alternative explanation relies on the excess 17-hydroxyprogesterone conversion to DHT through the “backdoor pathway (**Figure 4**) (22). Interestingly, after birth, circulating androgen levels are low or normal, therefore, virilization in these patients does not progress (58).

Data on pubertal development in these patients is scarce. One study reported pubertal status in seven patients with POR deficiency: most female patients presented with significant pubertal impairment, hypergonadotropic hypogonadism and ovarian cysts, prone to rupture. Potential underlying mechanism of the cysts formation was an excessive LH-mediated ovarian stimulation as a consequence of primary hypogonadism (62).

Specific *POR* mutations can result in a phenotype similar to the Antley–Bixler syndrome (MIM 201750) in both sexes, characterized by craniosynostosis, brachycephaly, midface hypoplasia, proptosis and choanal stenosis, radio-humeral or radio-ulnar synostosis, bowed femora and arachnodactyly (60).

##### 2.3.2.2 Diagnosis

Diagnosis of POR deficiency relies on the detection of a combined impairment of CYP21A2 and CYP17A1 activities, resulting in a combined mild elevation of pregnenolone, progesterone, 17-hydroxypregnenolone, 17-OHP and DOC, with variable cortisol response to ACTH. Genetic testing is usually needed to confirm the diagnosis (16, 60).

### 2.4 The Role of 11-Oxygenated Androgens in Hyperandrogenic Adrenal Disorders

An increasing interest has recently developed on the role of 11-oxygenated androgens (11-oxyandrogens) in hyperandrogenic adrenal disorders, especially CAH. 11-oxyandrogens are 19-carbon steroids primarily synthesized in the adrenal cortex: 11-hydroxyandrostenedione and 11-hydroxytestosterone are products of 11β-hydroxylase (CYP11B1) activity (**Figure 4**). On the other hand, 11-ketoandrostenedione and 11-ketotestosterone are produced in the kidneys from 11-hydroxyandrostenedione by 11β-hydroxysteroid dehydrogenase type 2 (63). The steroid 11-ketotestosterone is a potent androgen receptor agonist, showing an androgenic activity similar to that of testosterone (64). The specificity of 11-ketotestosterone as a biomarker of adrenal function is supported by the existence of higher concentrations in the adrenal vein than in the periphery (21), its rise during adrenarche (65) and after ACTH stimulation (66), and its complete decline in patients with adrenal insufficiency (21). In patients with CAH, high levels of 11-oxyandrogens correlate with adrenal volume and testicular adrenal rest tumors (67), and are particularly useful in the management of patients with discrepant 17-hydroxypreogesterone and androstenedione levels (63). On the other hand, 11-oxygenated androgens are not elevated in CAH due to 11β-hydroxylase or 3β-hydroxysteroid dehydrogenase deficiencies (18, 21).

## 3 Management of Patients With DSD Associated to Adrenal Dysfunction

The management of patients with DSD associated with adrenal dysfunction involves two main aspects: those related with genital and reproductive issues and those derived from the pathogenesis, frequently associated with adrenal insufficiency and steroid disorders.

### 3.1 Management of Genital and Reproductive Issues

Despite the significant societal changes observed in the last years vis-à-vis the importance of the sex of the newborn, gender assignment is still one of the major issues in patients with ambiguous genitalia. Decisions about the sex of rearing in babies with DSD can be particularly challenging, even if there is a growing comprehension that gender identity later in life may not correlate with the genetic, gonadal or genital sex of an individual. The karyotype and the degree of virilization are major drivers in the decision (68, 69). As already discussed, in the case of 46,XY DSD, the most severe forms of androgen deficiency result in completely female external genitalia, thus these individuals are assigned as girls. Conversely, those with less severe steroidogenic defects resulting in genital undervirilization are more frequently assigned male, given their good response to androgen replacement therapy. On the other hand, there is almost univocal consensus that newborns with 46,XX DSD benefit from female sex assignment (68), except for those with completely virilized external genitalia, where the decision may be controversial (69, 70).

#### 3.1.1 46,XY Patients

Patients with completely female external genitalia, reared as girls, do not require any treatment of their genitalia before pubertal age. The extirpation of the testes, usually present in abdominal position, is most often performed despite the lack of information about their malignant transformation potential, to avoid virilization at pubertal age. Estrogen replacement is necessary to provoke breast development, pubertal growth spurt and adequate bone mineralization. The vagina is generally shorter than normal because its upper part derives from the Müllerian ducts that regress in fetal life due to AMH action. This may cause discomfort for sexual intercourse in the adolescent, but surgical procedures may prove challenging. The absence of uterus leads to permanent amenorrhea and impossibility of gestation. However, the recent development of sophisticated surgical procedures allowing uterine transplantation in young women (71) and oocyte donation give hope to those who do not consider adoption.

In undervirilized boys, surgical correction of hypospadias and cryptorchidism is usually performed in infancy. Although some androgenic activity may be conserved, testosterone therapy is most frequently needed in order to support an adequate development of secondary sexual characteristics, growth and muscle and bone trophism. These patients are generally infertile: azoospermia results from insufficient intratesticular testosterone concentrations, which cannot be improved by exogenous testosterone treatment (72).

#### 3.1.2 46,XX Patients

Historical practice characterized by surgery in infancy, including clitoroplasty, vaginoplasty and urogenital sinus division, has raised controversy in the past years (15, 18). Unfortunately, little evidence exists regarding long-term sexual function outcomes, owing to the lack of controlled studies with adequate design. Expert opinion recommends that parents should be clearly informed about surgical options, including delayed surgery (26, 69). Urinary disorders, with frequent infections, may require early surgery; otherwise, the decision may be delayed until the patient can participate. Special attention should receive the examination of the genital anatomy to determine whether adequate menstrual flow will require surgery before pubertal onset. At the age of puberty, besides corticoid replacement, estrogen therapy may be needed to induce breast development and bone maturation and mineralization in 46,XX patients with 3βHSD2D or PORD.

Because anxiety, substance abuse and gender dysphoria are more frequently observed in association with fetal and postnatal excessive exposure to androgens, which results in impaired reproductive outcomes (23), psychological support is important. In women desiring conception, progesterone levels should be below 0.6 ng/ml (or 2 nmol/l), which can be attained with the administration of adequate doses of hydrocortisone or prednisolone, but not dexamethasone, which crosses the placenta and reaches the fetus (26). Successful pregnancy has been reported in 46,XX patients with CAH due to 21OHD treated with 1-2 mg of prednisolone at bedtime (73). In patients with 11βOHD, spironolactone used for the treatment of hypertension should be discontinued, due to its teratogenic potential (19).

### 3.2 Management of Adrenal Steroidogenic Dysfunction

Glucocorticoid and frequently also mineralocorticoid therapy is needed to replace adrenal cortical insufficiency, as well as to reestablish the physiology of the hypothalamic-pituitary-gonadal axis disrupted by the androgen excess in the most frequent forms of DSD of adrenal origin.

#### 3.2.1 Conventional Treatment

Glucocorticoid use for the treatment of CAH was introduced in the early 1950s by Wilkins, who was also the first to report that cortisone was able to suppress the elevated adrenal androgens (74). Since then, there has been little development in the way steroid hormone replacement therapy is conducted.

Glucocorticoids are currently the standard treatment for CAH associated to 9α-fludrocortisone, in cases of mineralocorticoid deficiency. A clinical practice guideline has recently been developed (26). The minimum dose that normalizes the excess of adrenal androgens and avoids cortisol insufficiency is recommended. Unfortunately, available preparations fail to suppress ACTH and to control adrenal androgen excess resulting often in glucocorticoid overtreatment (15). Therefore, management of CAH involves a challenging balance between glucocorticoid deficiency and hyperandrogenism, on one side, and hypercortisolism on the other, leading to short stature, obesity, hypertension, osteoporosis, and an adverse metabolic profile.

In growing children, hydrocortisone is the glucocorticoid of choice due to its short life, which allows childhood growth optimization. Recommended dose is 8-15 mg/m^2^ daily divided in three doses (14, 26). However, in late adolescence and adults, there are no standard clinical guidelines for glucocorticoid therapy and multiple preparations are available. Patients are generally switched to intermediate-acting glucocorticoids, such as prednisolone at 5.0–7.5 mg/day divided in two doses or long-acting glucocorticoids, such as dexamethasone at 0.25–0.50 mg at bedtime to improve compliance (75).

Mineralocorticoid supplementation with 9α-fludrocortisone is necessary in patients with aldosterone deficiency, present in different degrees in approximately 75-90% of patients with DSD of adrenal origin. All newborn patients detected by neonatal screening programs receive 9α-fludrocortisone, typically 100-200 μg/day divided in 1-2 doses. Sodium chloride supplements are recommended usually along the first years of life. In childhood, fludrocortisone doses usually range between 50-200 μg/day. Due to its prolonged half-life (18-36 hours) low doses can be administered once a day, although doses above 200 μg/day may still be divided to be given twice daily (14).

#### 3.2.2 Novel Treatment Options

Even though an adequate hormonal replacement would minimize complications and assure a normal quality of life, current therapies have failed to prevent co-morbidities, and adrenal crises still occur as a leading cause of death (15, 18, 76). This is partly due to the lack of adequate preparations, making it difficult to control the disease. For this reason, novel therapeutic options have been developed, and several clinical trials in adults and children are currently ongoing.

##### 3.2.2.1 Modified-Release Hydrocortisone

Chronocort^®^ is a modified release formulation of hydrocortisone (MR-HC) designed to mimic physiological cortisol secretion. Made of uniform multiparticulate beads with an inert core, a hydrocortisone drug layer and a delayed release enteric outer coat, it aims to mimic the cortisol circadian rhythm and control the overnight ACTH surge that leads to the increase androgens (75, 77, 78). MR-HC has shown to successfully lower androgen levels in patients and decrease the hydrocortisone equivalent dose. The larger evening dose reaches its peak in the early morning hours, and smaller morning dose peaks in the afternoon/evening thus providing glucocorticoid cover for the day with a more physiological cortisol profile. Chronocort^®^ is currently under regulatory review for the treatment of adults with CAH (18).

##### 3.2.2.2 Nevanimibe

Nevanimibe hydrochloride (ATR-101) inhibits acyl-coenzyme A: cholesterol O-acyltransferase (ACAT1/sterol O-acyltransferase 1 (SOAT1)), the main enzyme that catalyzes the esterification of free cholesterol to cholesteryl esters for storage in adrenal cortex cells. At lower concentrations, Nevanimibe selectively blocks adrenal cortex function of all three steroidogenic pathways (mineralocorticoid, glucocorticoid, and androgens). In a phase II study, single-blind, multicentric, placebo-controlled study of adults with classic 21OHD, Nevanimibe given orally decreased 17-hydroxyprogesterone levels within 2 weeks of treatment in most patients. However, it failed to effectively suppress androstenedione levels, a more durable measure of adrenal control (79). This therapy would allow the use of lower glucocorticoid doses, minimizing adverse events as compared to standard therapy and might represent a promising addition to current treatment strategies. However, larger long-term studies with higher dose are needed to evaluate safety and efficacy.

##### 3.2.2.3 Abiraterone

Abiraterone acetate (AA) is a prodrug that is metabolized to abiraterone, a potent CYP17A1 inhibitor. It is used to suppress circulating testosterone in the treatment of prostate cancer improving survival rates. As P450c17 activity is needed for the synthesis of all androgen, it has been hypothesized that by inhibiting it with abiraterone acetate, added to stable doses of physiological hydrocortisone and 9α-fludrocortisone acetate, androgen excess present in 21OHD might be controlled, thus eliminating the need for supraphysiological glucocorticoids doses (80). In a phase I study of adult women with inadequately controlled classic 21OHD, abiraterone acetate added to hydrocortisone was able to normalize androstenedione on days 6 and 7 in at least 80% of participants without causing hypertension or hypokalemia. In a recent study, abiraterone acetate has shown to effectively and consistently lower 11-oxygenated androgens in 21OHD (81). Abiraterone acetate might also be beneficial to suppress androgens and estrogens in prepubertal children with classic CAH until the anticipated age of puberty. A phase I trial testing this approach is underway (NCT02574910).

##### 3.2.2.4 Corticotropin-Releasing Factor Receptor-1 (CRF1R) Antagonists

Corticotropin-Releasing Factor (CRF) is released from the hypothalamus into the hypophyseal portal system, acting directly on specific receptors on pituitary corticotropes. CRF type 1 (CRF1R), one of the two CRF receptors, is especially abundant in the pituitary and in the neocortex. CRF receptor antagonists reduce ACTH and adrenal steroid production. A phase Ib study including 8 women with classic CAH showed that the CRF1R antagonist NBI-77860 can effectively decrease the early morning rise of ACTH and 17-hydroxyprogesterone, eliminating the need for supraphysiologic doses of glucocorticoids (82). Tildacerfont (SPR001; LY2371712) is a second generation CRF1R antagonist that binds to pituitary receptors with high affinity, thus decreasing ACTH secretion. In two recent phase 2 clinical trials including adult patients with CAH, oral tildacerfont reduced ACTH, 17-hydroxyprogesterone and androstenedione for up to 12 weeks; normalization of ACTH and androstenedione was achieved in 40-60% of the patients according to dosage (83). Longer term multidose trials are needed to determine safety and effectiveness of this potential therapy.

#### 3.2.3 Potential Options Based on Cell- or Gene-Therapy

Cellular reprogramming and gene therapy are theoretically viable options that are under current investigation. Different cell types of mouse or human origin have been used for cellular reprogramming to an adrenocortical phenotype, showing ultrastructural features typical of steroidogenic cells, expression of steroidogenic enzymes and secretion of steroids in response to ACTH. The reprogrammed human steroidogenic cells were viable when experimentally transplanted into the kidney capsule or in the adrenals of mice (84). These cells could be helpful to model adrenal defects and represent a potential therapy strategy.

An alternative strategy is gene therapy, which was tested in mice with a deletion encompassing the *Cyp21* locus who received an injection of a replication-deficient adenovirus containing the mouse gene extra-adrenally (85), or the human *CYP21A2* gene intra-adrenally (86) or intravenously (87). In all cases the adrenal function was restored, giving hope for the development of gene therapy in humans with CAH due to 21OHD.

## 4 Concluding Remarks

Disorders of adrenal function are the major cause of DSD. In 46,XX patients, it represents more than 90% of the underlying etiologies. In 46,XY individuals, the associated gonadal steroidogenic failure leads to undervirilization and ambiguous genitalia. DSD associated with adrenal dysfunction represent a challenging condition, given the risk of life associated with adrenal failure. Management requires a balanced supplementation of glucocorticoids –and mineralocorticoids in almost 75% of the cases–, together with consideration of the genital and reproductive disorders. Unfortunately, despite the long-lasting awareness of these conditions, evidence-based recommendations are still scarce, and adequately designed studies need to be carried out in order to provide a better standard of care for these relatively frequent disorders.

## Author Contributions

All authors listed have made a substantial, direct and intellectual contribution to the work, and approved it for publication.

## Conflict of Interest

GF is currently employed by Takeda Pharma S.A.

The remaining authors declare that the research was conducted in the absence of any commercial or financial relationships that could be construed as a potential conflict of interest.

## Publisher’s Note

All claims expressed in this article are solely those of the authors and do not necessarily represent those of their affiliated organizations, or those of the publisher, the editors and the reviewers. Any product that may be evaluated in this article, or claim that may be made by its manufacturer, is not guaranteed or endorsed by the publisher.
